# Reversible mechanical control of molecular conformation

**DOI:** 10.1038/s42004-020-00389-8

**Published:** 2020-10-26

**Authors:** Andrew J. Bissette

**Affiliations:** Communications Chemistry, https://www.nature.com/commschem

## Abstract

Controlling molecular conformation through macroscopic mechanical stimulus may have applications in chiroptical devices, but achieving this in a 3D material is challenging. Now, a quantitative relationship between stretching of an elastomer and reversible conformational changes of a crosslinked molecule has been established.

Transferring information about molecular conformation to the macroscopic scale can allow remarkable control over the shape and properties of synthetic materials. The opposite process—using macroscopic mechanical stimuli to reversibly tune molecular conformation—is less well understood. Now, a team of researchers from the University of Strasbourg and the University of Lyon led by Loïc Jierry show that stretching of an elastomer allows for reversible and precise control over the dihedral angle of an axially chiral dopant. (10.1002/anie.202010604).

Controlling molecular conformation through macroscopic mechanical stimulus has been achieved previously in other systems, particularly in crystals and self-assembled monolayers. These provide excellent models to establish the concept owing to their highly ordered structures, allowing changes in conformation to be readily detected through routine spectroscopy. This can be achieved by embedding a chiral molecule within the material and monitoring changes in its circular dichroism spectrum, which reflect changes in conformation. For example, Ariga and coworkers previously induced a reversible 10 degree change in the dihedral angle of a binaphthyl derivative (BINOL) by compressing a monolayer^1^. But both crystals and monolayers have limitations which may hinder real-world applications. The rigidity of crystals limits the magnitude of deformation, while the fragility of monolayers precludes more challenging manipulations. Applications of mechanochemically controlled conformational switching likely require a more robust substrate.

Three dimensional polymer networks offer a balance of flexibility and strength which may aid future applications, but impose their own limitations. “The main challenge is to our ability to measure conformational changes through a polymer network, which was not an easy task”, says Jierry, “With the optical signals related to chirality being much lower than those related to the polymer network stretching (10^3^ lower), the difficulty is to distinguish the meaningful signals from the false ones”. Conformational changes in the disordered polymer network during mechanical stimulus confound simple efforts to monitor the conformation of chiral dopants. Furthermore, the lack of an ordered lattice means the dopant molecules are randomly oriented at rest, complicating analysis.

To solve this problem, the authors doubly-crosslinked a single-enantiomer BINOL derivative within a polydimethylsiloxane (PDMS) elastomer and studied it using a custom sample holder (Fig. 1, ref. ^2^). This allowed optical measurements to be taken at specific angles, thereby decoupling the confounding contributions arising from the polymer network from the circular dichroism signal arising from the chiral BINOL molecules. “The real circular dichroism signal is corrected from the different linear contributions and unambiguously related to the conformation change of the chiral molecule under stretch”, explain the authors.

**Fig. 1:**
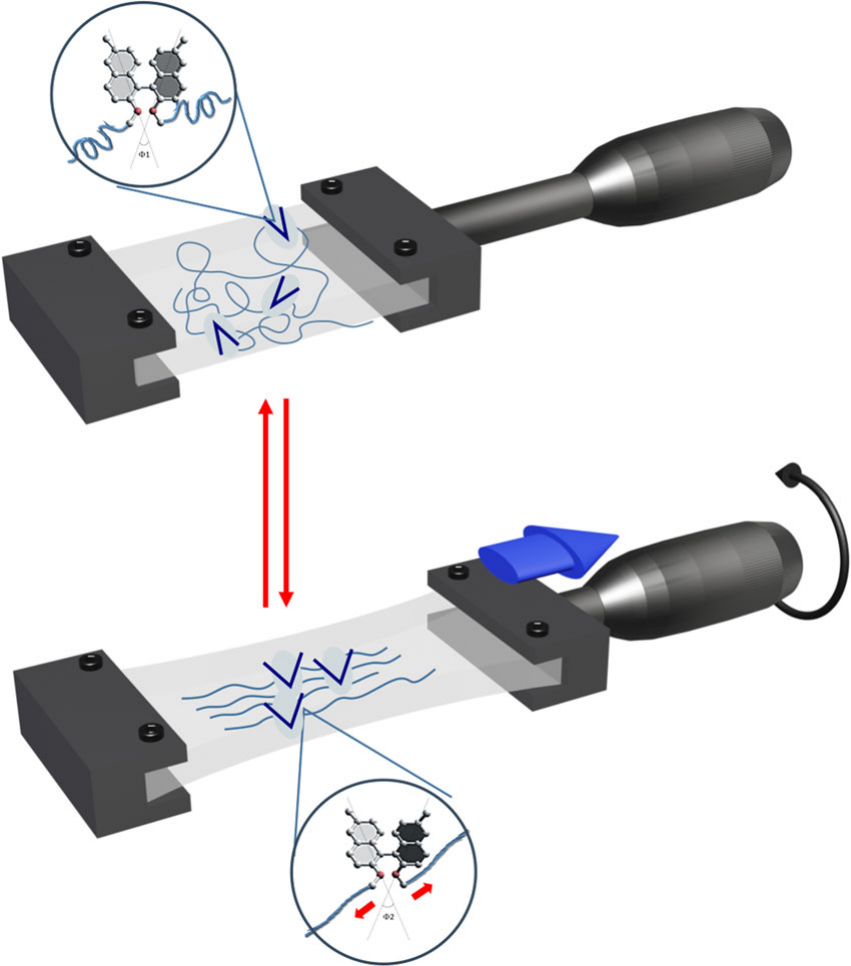
Reversible mechanochemical control of molecular conformation. Stretching and relaxation of a PDMS elastomer causes reversible control over the dihedral angle of crosslinked BINOL molecules. Use of a custom sample holder allows the dihedral angle to be monitored quantitatively without confounding optical contributions from the surrounding elastomer^2^. Copyright Wiley-VCH GmbH. Reproduced with permission.

This approach enabled the authors to continuously monitor the dihedral angle of the BINOL monomers during reversible mechanical stretching and relaxation. The use of a robust elastomer allowed over 20 cycles of stretching and relaxation at different degrees of extension to be performed on a single sample, with complete recovery of the initial circular dichroism signal at rest between cycles. Computational modeling correlated the obtained signal with the dihedral angle at BINOL as well as calculation of the forces involved: stretching of the elastomer increases the dihedral angle, reaching a maximum increase of around 20 degrees at 170% extension, corresponding to around 175 pN.

The study provides a compelling example of the robust and repeatable transfer of mechanochemical stimulus on the macroscopic scale to conformational changes in a chiral dopant. Beyond fundamental interest, the findings may have applications in the study of chiroptical materials that rely on axially chiral molecules for function.
